# Accuracy and reproducibility of somatic point mutation calling in clinical-type targeted sequencing data

**DOI:** 10.1186/s12920-020-00803-z

**Published:** 2020-10-15

**Authors:** Ali Karimnezhad, Gareth A. Palidwor, Kednapa Thavorn, David J. Stewart, Pearl A. Campbell, Bryan Lo, Theodore J. Perkins

**Affiliations:** 1 Ottawa Hospital Research Institute, 501 Smyth Road, Ottawa, K1H8L6 Canada; 2 Department of Biochemistry, Microbiology and Immunology, University of Ottawa, 451 Smyth Road, Ottawa, K1H8M5 Canada; 3 School of Epidemiology and Public Health, University of Ottawa, 600 Peter Morand Crescent, Ottawa, K1G5Z3 Canada; 4 The Ottawa Hospital, 501 Smyth Road, Ottawa, K1H8L6 Canada

**Keywords:** Cancer genomics, Clinical genomics, Variant calling, Single nucleotide variants, High-throughput sequencing, Somatic point mutations, Personalized medicine

## Abstract

**Background:**

Treating cancer depends in part on identifying the mutations driving each patient’s disease. Many clinical laboratories are adopting high-throughput sequencing for assaying patients’ tumours, applying targeted panels to formalin-fixed paraffin-embedded tumour tissues to detect clinically-relevant mutations. While there have been some benchmarking and best practices studies of this scenario, much variant calling work focuses on whole-genome or whole-exome studies, with fresh or fresh-frozen tissue. Thus, definitive guidance on best choices for sequencing platforms, sequencing strategies, and variant calling for clinical variant detection is still being developed.

**Methods:**

Because ground truth for clinical specimens is rarely known, we used the well-characterized Coriell cell lines GM12878 and GM12877 to generate data. We prepared samples to mimic as closely as possible clinical biopsies, including formalin fixation and paraffin embedding. We evaluated two well-known targeted sequencing panels, Illumina’s TruSight 170 hybrid-capture panel and the amplification-based Oncomine Focus panel. Sequencing was performed on an Illumina NextSeq500 and an Ion Torrent PGM respectively. We performed multiple replicates of each assay, to test reproducibility. Finally, we applied four different freely-available somatic single-nucleotide variant (SNV) callers to the data, along with the vendor-recommended callers for each sequencing platform.

**Results:**

We did not observe major differences in variant calling success within the regions that each panel covers, but there were substantial differences between callers. All had high sensitivity for true SNVs, but numerous and non-overlapping false positives. Overriding certain default parameters to make them consistent between callers substantially reduced discrepancies, but still resulted in high false positive rates. Intersecting results from multiple replicates or from different variant callers eliminated most false positives, while maintaining sensitivity.

**Conclusions:**

Reproducibility and accuracy of targeted clinical sequencing results depend less on sequencing platform and panel than on variability between replicates and downstream bioinformatics. Differences in variant callers’ default parameters are a greater influence on algorithm disagreement than other differences between the algorithms. Contrary to typical clinical practice, we recommend employing multiple variant calling pipelines and/or analyzing replicate samples, as this greatly decreases false positive calls.

## Background

Next generation sequencing (NGS) technologies have been used to catalogue genetic mutations in cancer [1]. Studies employing NGS have identified specific genetic mutations that reliably predict therapeutic success with targeted treatment regimens in many forms of cancer [2], including non-small cell lung cancer, which is our long-term focus. Importantly, patients with oncogenic driver mutations have better tumour control with targeted agents than with chemotherapy, while those lacking such a mutation derive more benefit from chemotherapy [3]. Thus, accurate identification of the mutated or non-mutated status of key genomic sites is critical for patient therapy.

Developments in NGS technologies are empowering the analyses of whole cancer genomes, providing insights into the task of somatic mutation calling [4] and have made it possible to characterize the genomic alterations in a tumour in an unbiased manner [5]. With the advancement of NGS technologies, the number of large-scale projects (especially cancer projects) dealing with somatic point mutation in various tumour types has been increasing rapidly. However, in clinical practice, there is only a limited number of actionable mutations that are of interest–those for which a specific therapy is recommended, or perhaps for which a clinical trial may be ongoing. In such cases, targeted sequencing is the preferred option, offering lower cost and higher coverage of areas of interest [6].

The majority of mutation callers available in the literature have been designed to analyze matched tumour-normal samples [7], as comparing the two helps discriminate cancer-related and non-cancer-related mutations. However, many clinical labs do not routinely acquire matched healthy tissue, so that variant calling must be performed on the tumour tissue only. As such, tumour-only mutation callers have also been developed. A few mutation callers such as MuTect2 and VarDict are versatile enough to analyze both matched tumour-normal and tumour-only samples. For a recent up-to-date list of mutation callers, readers may refer to [7], where 46 programs are reviewed. In this study, we focus on tumour-only mutation calling.

A typical workflow of mutation calling can be divided into three steps. First, reads are processed and low-quality bases and any exogenous sequences such as sequencing adapters are excluded from the reads. This can be performed by using tools such as Cutadapt [8] and NGS QC Toolkit [9]. Second, the cleaned reads are mapped to a reference genome. This base-to-base alignment can be done by using common tools such as BWA aligner [10] for DNA sequencing or TopHat [11] for RNA sequencing. The last step of the process is to separate real mutations from artifacts that might be present due to a process of library preparation, read errors, mapping errors, and so on. Some mutation callers perform the above three-step process as a built-in procedure (see [7]), while others start from the point of cleaned, mapped reads.

Several analysis packages with different algorithms have been introduced in recent years to increase mutation detection accuracy. Significant discrepancies between the results of different algorithms have been observed, which leads to a difficulty in selecting candidate mutations for validation [12]. Such disagreement seems to partially root from different error models and assumptions made in each algorithm. In addition, various sources of errors such as sequencing errors and read alignment errors make the process more challenging. Thus, detection accuracy remains questionable and in fact, has become a major challenge.

Previous benchmarking studies offer some guidance in this regard, each having its strengths and weaknesses. For example, Spencer et al. [13] evaluated four mutation callers on a compendium of data from several cell lines and mixtures of DNA from those cell lines—artificially “creating” mutations at different allele frequencies. However, they focused on only one targeting panel, the Washington University comprehensive cancer set WUCaMP27 version 1.0, and one sequencing platform, the Illumina HiSeq 2000. Also problematically, they defined gold standard mutations by intersecting variant calls from GATK and SAMtools, two of the four programs they benchmarked. Derryberry et al. [14] focused on the technical reproducibility of variant calls, studying 55 replicate pairs of data from gliobastoma tumours. However, this was whole genome sequencing data, where coverage was substantially lower than for targeted panels. Xu et al. [15] performed a thorough comparison of mutation callers on exome sequencing data of pure and admixed cell lines, where gold-standard mutations were established by prior independent work. Moreover, they explored, to some extent, the effects of varying variant-calling thresholds. However, their work was limited to tumour-normal variant calling. Numerous other benchmarking studies have been performed; we mention these only as examples. Of note, new “best practices” for benchmarking variant calling have recently been proposed [16].

Of course, no single study can exhaustively address all possible relevant issues in variant calling. We seek here to add to the conversation by comparing multiple variant callers, on data from two cell lines, sequenced on two different platforms using two different targeted panels, in multiple biological and technical replicates. We minimize circularity in defining gold-standard mutations by relying on previous high-quality work using independent data and variant calling methods. We study agreements and disagreements in variant calls, depending on algorithms, algorithm settings, and between replicates. Our key findings are: that sequencing platforms are not a major influence on variant calling; that variant callers can disagree wildly when used with default/recommended settings, but that they agree much more when settings are made consistent; and that intersecting the results from different algorithms (which is not unusual in research practice, though possibly unusual in clinical practice) or from replicate samples (which is definitely unusual in clinical practice), can greatly reduce false positive calls while maintaining sensitivity for detecting genuine mutations.

## Methods

**Cell lines and DNA sequencing data generation:** Two commercially-available cell lines, with catalog IDs GM12878 and GM12877, were obtained from Coriell Cell Repositories in April 2017. Cells were prepared as standard for targeted clinical sequencing. Briefly, cells were expanded as per the ATCC recommended protocol, harvested, and processed into FFPE cytoblocks. DNA and RNA were isolated for each cell line using minimum of 6 x 10 *μ*m sections and AllPrep DNA/RNA FFPE Kit (Qiagen) according to vendor’s recommended protocol. Nucleic acid quality assessment and quantitation were respectively performed using the Fragment Analyzer (AATI) and Qubit (Thermo Fisher Scientific). Three such biological replicates were prepared for each cell line. Two of those biological replicates were sequenced in technical triplicate using the TruSight 170 (Illumina) approach, while one biological replicate was sequenced in technical triplicate using an Oncomine Focus (Thermo Fisher Scientific) approach. In total, this produced our 18 datasets = 2 cell lines ×3 biological replicates ×3 technical replicates. TruSight 170 libraries were prepared using 40 ng DNA or RNA input. Oncomine Focus libraries were prepared using 10 ng DNA or RNA as input. Illumina libraries were sequenced on the NextSeq 500, with 8 RNA and DNA libraries pooled in each High Output 300 cycle run. Oncomine Focus libraries were sequenced on the Ion Torrent, with 6 RNA and DNA libraries pooled on each 318 chip. The Illumina TST170 assay provides full exonic coverage for 170 cancer-associated genes, covering 527,121 total bases, with 3,064 genomic intervals. The Oncomine Focus panel covers 29,008 total bases in 47 genes, using 269 genomic intervals. In this study, we use only the DNA sequencing data from either panel.

**DNA sequencing data processing:** TruSight sequencing data in FASTQ format was quality checked and mapped to the hg19 genome (version GRCh37 obtained from UCSC Genome Browser) using the recommended BaseSpace pipeline, which relies on Isaac DNA aligner v3.16.02.19. For the Oncomine data, BAM files were obtained from the Ion Torrent online workflow. But because of indexing issues, reads were extracted from the BAM files back into FASTQ format using Picard v2.10.7. Then the reads were mapped to hg19 using BWA aligner version v0.7.17-r1188, producing the BAM files to be used for variant calling. All FASTQ files are available from SRA under accession PRJNA614006.

**Single nucleotide variant calling:** We evaluated six software packages capable of single nucleotide variant (SNV) calling: SAMtools v1.9 [17], VarScan2 v2.3.9 [18], MuTect2 v4.beta.3-SNAPSHOT [19], VarDict [20], Pisces v5.2.0.1 [21], and the Ion Torrent Variant Caller (ITVC) [22]. All versions were the most recent available at the time of our study. With the exception of ITVC, we downloaded all software packages and installed them on our local compute cluster. Each takes as input BAM files of mapped reads or pileups computed from BAM files, e.g. by the mpileup function of SAMtools. Each was used to call variants in tumor-only mode, meaning mutations were called relative to the hg19 genome. Each program outputs some form of variant call file (VCF), from which we extracted SNV calls along with associated information provided by the variant caller, such as depth of coverage, alternative allele frequency, p-value, etc. For ITVC, the results on the Oncomine Focus data were obtained through their web-based analysis tool.

Each variant caller has some pre-defined but adjustable parameters, such as: minimum variant frequency, minimum coverage, minimum base quality score and minimum mapping quality score. In our initial tests, we ran each software with its default, recommended parameters. Table 1 summarizes key parameters and their default values for different programs. In later testing, we made the parameters of different algorithms to be as similar as possible. Specifically, we set the minimum variant allele frequence to 0.01, the minimum read depth for variant calling to 10 reads, the minimum base call quality to 20, and the minimum read mapping quality to 20.

**Table 1:** Various parameters along with their default values defined in each mutation caller

**Option**	**MuTect2**	**VarScan2**	**SAMtools**	**VarDict**	**Pisces**
Threshold for allele frequency	–	0.01	–	0.05	0.01
Base quality score threshold	18	–	–	–	–
Max base quality score	–	–	–	–	100
Min map quality	20	null	0	null	1
Mean map quality	–	null	–	null	–
Max map quality	null	–	–	–	–
Min coverage	–	8	–	–	10
Max coverage	–	–	250	–	–
Min supporting variant reads	–	2	–	–	–
Min variant quality score	–	–	–	–	20
Strand bias filter	–	–	–	–	0.5
Min reads to strand bias	2	–	–	–	–

A dash means that the corresponding parameter was not defined in the caller’s settings

**Performance assessment:** For the GM12878 and GM12877 cell lines, we obtained gold-standard variant calls from [23]. We assumed that these and only these SNVs should be present in our cell lines. We intersected the SNVs provided by that study with the genomic intervals covered by the TruSight170 or Oncomine Focus panels, to determine which should be detected. If a caller identifies one of these mutations in a particular dataset, it is designated a true positive (TP). If a mutation within the genomic intervals for a panel is not identified by a caller from that dataset, it is designated a false negative (FN). Any called mutation that falls within the genomic regions but that is not on the gold-standard list is considered a false positive (FP). Variant callers are compared based on their TP, FN, FP numbers and scores derived from these, particularly precision = TP / (TP+FP) and sensitivity = TP / (TP+FN).

## Results

For the purpose of benchmarking different variant callers, we used clinical sample handling protocols to perform targeted DNA or DNA/RNA sequencing on multiple biological replicates of multiple cell lines. Because accuracy assessment requires us to know which mutations should be present in the samples, we selected two very well characterized Coriel Cell lines, GM12878 and GM12877. As our long-term goal is understanding best methods for variant calling in formalin-fixed, paraffin-embedded (FFPE) lung cancer fine-needle aspirates (FNAs), we performed independent preparations of the GM12878 and GM128977 cells for analysis in FFPE cytoblocks. We selected two targeted assays for testing. One was the TruSight170 Tumor Assay (TST170) from Illumina. TST170 is a comprehensive RNA/DNA hybrid-capture assay that provides full coverage for 170 solid tumor-associated genes. It is amenable to testing on FFPE samples of relatively limited sample availability. This assay tests for the presence of multiple classes of structural variants that are relevant to clinical diagnostics including SNVs, fusions, copy number variation, and INDELs. Here, however, we will focus only on the SNVs. The TST170 sequencing was performed on an Illumina NextSeq500 sequencer. We also tested the Oncomine Focus panel, which again is amenable to FFPE samples with limited material. Oncomine Focus is an amplification-based panel covering 47 genes, and sequencing was performed on an Ion Torrent PGM. The sequencing data is available through SRA under accession PRJNA614006. Table 2 summarizes several key features of the sequencing data we generated. For the TST170 panel, read depths varied from approximately 2.4 million to over 13 million per sample. Within the genomic regions covered by the panel, this resulted in average coverage levels ranging from 203 to over 1000 reads per basepair. For the Oncomine data, read depths ranged from 1.3 million to over 15 million, resulting in coverages ranging from 415 to over 4000 reads per basepair.

**Table 2:** Summary of read depth and coverage (average reads covering each basepair covered by the panel) for TruSight Tumor 170 (TST170) and Oncomine Focus (OF) panels

	**TST170 - 12878**		**TST170 - 12877**		**OF - 12878**		**OF - 12877**	
**Rep**	**Reads**	**Cover**	**Reads**	**Cover**	**Reads**	**Cover**	**Reads**	**Cover**
1	13275831	1056	3526629	300	835719	2437	602628	1829
2	7598224	659	3285901	280	213469	625	769173	2252
3	8279644	725	2440647	203	138350	415	1566916	4338
4	7435784	640	3640546	323	–	–	–	–
5	7519631	648	4731704	412	–	–	–	–
6	7775631	672	4101646	366	–	–	–	–

### Sequencing data shows expected SNVs in GM12878 and GM12877 cell lines

Recent work has established a comprehensive and genome-wide catalog of high-confidence variants for a collection of Coriell cell lines, including GM12878 and GM12877 [23]. That work relied on whole-genome sequencing data of 17 individuals in a three-generation pedigree, and variant calling using four programs: FreeBayes [24], Platypus [25], GATK3 [26] and Strelka [27]. By intersecting the locations of these known mutations with the TST170 genomic regions, we determined that our GM12878 and GM12877 data should contain 343 and 336 of these mutations, respectively. Intersecting with the Oncomine Focus genomic regions, we predicted that 26 and 24 known mutations should appear in GM12878 and GM12877 data respectively.

**Fig. 1:**
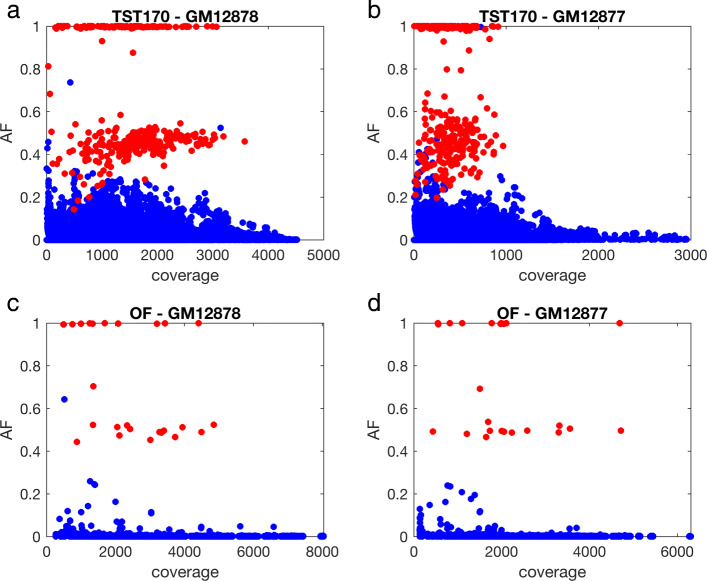
Allele frequency (AF) and coverage of gold-standard mutations (red) and reference sites (blue) in replicate one of our data for each of four conditions: (**a**) GM12878 cells sequenced using TruSight170 panel; (**b**) GM12877 cells sequenced using TruSight170 panel; (**c**) GM12878 cells sequenced using Oncomine Focus panel; (**d**) GM12877 cells sequenced using Oncomine Focus panel

The first thing we checked was whether there was indeed evidence for these high-confidence, gold-standard mutations in our data. While the ability to call a variant depends on many factors, two key factors are: (1) the coverage (or read depth) at the site, and (2) the alternative allele frequency (AF), which is the fraction of reads showing a non-reference nucleotide at a site. We mapped all read data to the hg19 genome (see Methods for details), and computed the coverage and alternative allele frequencies at all sites covered by the panels using Bam-readcount (https://github.com/genome/bam-readcount). This includes both positions where we expect the GM12878 and GM12877 cell lines to different from hg19, and positions that we expect to be the same. Figure 1 shows the results for the first replicate in each of our four conditions, with red spots indicating sites believed to have SNV compared to hg19, and blue spots indicating sites believed to have the reference nucleotide.

Figure 1a, for example, shows AF and coverage of our TST170 data from the GM12878 cell line. We observe three main clusters of points in the plot. One cluster is at or very near AF = 1, and appears to consist of homozygous sites on our gold-standard list of mutations relative to hg19, as indicated by the red color. A second cluster centers near AF = 0.5, but with substantially greater spread, and predominantly consists of heterozygous mutation sites (also in red). As would be expected on simple random-sampling grounds, there is more spread in AF around AF = 0.5 when read depth is lower, with some heterozygous variants having AF below 0.2 and others over 0.8. The third cluster of points centers near AF = 0, and primarily consists of homozygous sites that match the hg19 reference. The non-zero allele frequencies here could, in principle, represent genuine SNVs present in cells. Their low AF argues against them being heterozygous or homozygous mutations, unless they are present in only a subset of our cells. In a later section we look at the reproducibility of apparent mutations in different replicates, and conclude this is not likely the case. Rather, these non-reference reads are likely artifacts, perhaps resulting from sequencing or mapping errors.

Figure 1b-d shows qualitatively similar results for our other three conditions—three point clusters, with the upper two largely consisting of known mutations, either homozygous or heterozygous. The TST170–GM12877 data in panel B shows greater spread in the AF ≈ 0.5 cluster. We suspect this is due in part to the lower overall read depths of these experiments, which came out at roughly half compared to GM12878. Few mutations exceed a coverage of 1000 reads in our GM12877 data, whereas that is the approximate median for our GM12878 data. It is not clear if this is the only factor leading to the greater AF spread. We also observe slightly greater frequency of mutations just below the level of AF=1. This may suggest a slightly higher per-base read error rate or possibly alignment errors. The OF data in panels C and D is distinct from the TST170 data primarily in that many fewer bases are covered. Therefore, there are many fewer points on the plots, and coverage levels are higher on average.

Figure 1 shows that, at least in replicate one of our four conditions, every gold-standard mutation has at least some support—in the sense of a non-zero AF. Indeed, across all 18 of our datasets, we found only a single gold-standard mutation site in replicate three of our TST170–GM12877 that had no supporting evidence whatsoever. Therefore, we can conclude that our data contains evidence of virtually all expected gold-standard mutations. This is not to imply that it is easy to distinguish those mutations from reference sites. As we can also see in the figure, some reference sites have coverage and AF comparable to some gold-standard mutations, and some gold-standard sites have quite low coverage and/or AF.

### Variant callers disagree greatly under default configurations

We investigated the performance of several high-profile variant callers in separating the gold-standard mutations from the non-mutated sites. For all the data sets, we tested the following four variant callers: MuTect2 [19], SAMtools [17], VarDict [20], and VarScan2 [28]. In addition, we tested the vendor-recommended variant caller on each dataset. For the TST170 data, generated on an Illumina sequencer, that means Pisces [21]. For the OF data, generated on an Ion Torrent sequencer, that means the Ion Torrent Variant Caller (ITVC) [22]. We could not apply ITVC to the Illumina-generated data, nor do we report on the Illumina-recommended caller, Pisces, on the Ion Torrent-generated data. In total then, five different variant callers were applied to each dataset. See Methods for exact version numbers and other details. These variant callers were chosen for different reasons. The motivation for testing the vendor recommended callers, Pisces and ITVC, is that these are the approaches that clinical labs would tend to use by default. The SAMtools package is one of the most highly cited programs in all of bioinformatics, and its variant-calling facilities in particular have been used widely. VarScan2 is another highly cited and well-established package for mutation calling. MuTect2 is the latest version of the MuTect program, which won a DREAM somatic genotyping contest [29]. VarDict is a more recent program and has facilities designed specifically for clinical-type sequencing protocols. Importantly, SAMtools, MuTect2, VarScan2, and VarDict also have several key properties that recommended them for our study: they are freely available to use; their code is open source, so we could install it on our local machines and compute cluster; they are capable of variant calling in tumor-only mode; and all output VCF files containing SNV calls that can readily be compared to our gold-standard mutation list and to each other.

**Fig. 2:**
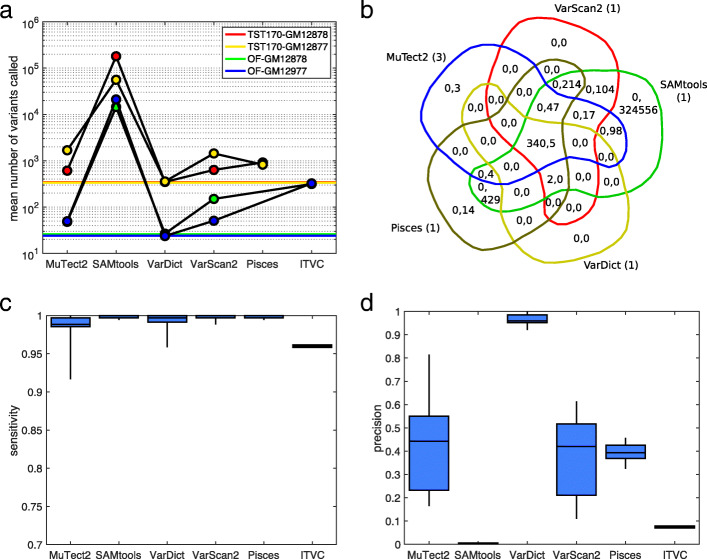
Summary of variant calling across all 18 datasets, when using *default/recommended* calling parameters. **a** Numbers of SNVs called by different variant callers, averaged across replicates within our four conditions: GM12878 or GM12877 cells, sequenced using TST170/Illumina or OF/IonTorrent. Colored horizontal lines indicate the number of gold standard mutations that ideally would be detected. **b** Venn diagram of calling results for five callers on replicate one of the TST170–GM12878 data. Within each region, the two numbers give the number of true positive (TP) and false positive (FP) calls agreed upon by the relevant algorithms. The numbers in parentheses next to each algorithm’s name are the number of false negatives (FN) by that algorithm. **c**,**d** Sensitivity (TP/TP+FN) and precision (TP/TP+FP) for each algorithm across all the datasets to which it was applied. The box-and-whisker plots show the percentiles: 0% (i.e. minimum), 25%, 50% (i.e. median), 75%, and 100% (i.e. maximum)

Some clinical sequencing centers have the expertise to carefully tune the bioinformatics tools they employ, but many simply use some established or recommended pipeline with default settings for all parameters and filters. We first compared the variant callers under default settings, as downloaded and/or recommended in their associated publications (see Methods for details). Figure 2 summarizes the results, while Supplementary Tables 1-4 give the exact numbers of calls, true positives, false positives, false negatives, sensitivity, and precision for every caller on every dataset.

Figure 2a shows the average number of mutations called by each variant caller in each of our four conditions: GM12878 or GM12877 cells, sequenced by TST170/Illumina or OF/Ion Torrent. Horizontal colored lines also indicate the correct number of mutations covered by the panels that would, ideally, be discovered. The various programs call wildly different numbers of SNVs from the same datasets. The most extreme is SAMtools, which calls over 100,000 SNVs on average in the TST170 data, and over 10,000 SNVs in the OF data. Considering that these panels cover 527,121 and 29,008 total bases respectively, this is an astonishingly high call rate. This happens because SAMtools’s default behavior is more or less to report anything that has any chance of being a mutation. Even a single read with a non-reference basepair is enough to cause SAMtools to flag a site. (We do not intend to criticize SAMtools here, but merely to highlight its default behaviour as being distinct from that of other programs.) With the depth of coverage in our data and even a relatively low per-base error rate, many positions end up with some non-reference reads. Pisces is the next most profligate caller, reporting on average approximately three times as many sites as in our gold-standard list. At the opposite end of the spectrum, VarDict appears quite strict, calling only a few more sites than expected from the gold standard. Indeed, it is the only variant caller whose default performance does not substantially overestimate the numbers of mutations in these cell lines, as represented in our data.

Beyond the mere numbers of SNVs called by each algorithm, it is important to understand which calls are correct and which are not. Again, Supplementary Tables 1-4 give such performance details. Figure 2b, however, gives a representative Venn diagram of SNV calls on replicate one of our TST170–GM12878 data. Within each region of the Venn diagram, which corresponds to a subset of the callers, the numbers of true positive (TP) and false positive (FP) calls are indicated. The numbers of false negative (FN) calls by each algorithm are indicated in parentheses next to their name around the outside of the Venn diagram. The good news is that all five variant callers correctly call almost all of the gold-standard mutations. All five variant callers agree on 340 of the 343 gold-standard mutations, and two more gold-standard mutations are called by all programs except MuTect2. All five programs miss one of the gold-standard mutations. The Venn diagram shows, as we can also deduce from panel A, that SAMtools calls a very large number of false positives. However, and perhaps surprisingly, there remains a small number of false positives called uniquely by other programs. Pisces calls SNVs at 14 sites not called by any other program, and MuTect2 calls three unique SNVs. VarScan2 does not call any unique false positives, although it shares 104 calls with SAMtools that are not called by any other program. VarDict reports only five false positives, and these five are also reported by every other program.

Figure 2c reports the sensitivity values (TP/TP+FN) for each algorithm across the datasets to which it is applied. As in panel B, we see that most algorithms are successful at identifying all or nearly all of the gold-standard mutations. The ITVC program is a bit of an exception, having sensitivity values around 96%, despite the fact that the average number of SNVs it calls is about 10 times higher than the number of true SNVs (Fig. 2a). For the other algorithms, sensitivity is higher than 98% on all except a few datasets, where MuTect2 and VarDict have some difficulties. As seen in Supplementary Tables 1-4, those problematic datasets are all OF/Ion Torrent datasets, where ITVC too has trouble.

Figure 2d reports the precision values (TP/TP+FP) for each algorithm. Precision is one minus the empirical false discovery rate (EFDR), defined as FP/TP+FP. As one would expect, the algorithms that call high numbers of SNVs necessarily have very poor precision—an abysmal <1% for SAMtools, and approximately 8% for ITVC. VarDict stands out as having quite high precision, at over 90% on all datasets, and averaging over 95%. The other algorithms have precision hovering around 40% to 50% on average, and thus EFDRs between 50% and 60%, which is higher than would likely be acceptable by most clinical sequencing centers.

### Choosing consistent filtering parameters makes variant calls more similar

Although each variant caller relies on a distinct statistical model, they also filter both inputs and results in various ways. Differences in the default parameters of these filters are an obvious possible explanation for divergent variant calls observed in the previous section. Thus, we re-analyzed all data with the same algorithms, but setting their filtering parameters to be as similar as possible (see Methods). We set the minimum alternative allele frequency for a called variant to 0.01, the minimum coverage to 10 reads, the minimum base call quality to 20, and the minimum mapping quality to 20. These are not restrictive parameter choices, aimed at eliminating false positives calls. Later, we will return to the question of whether more restrictive filtering can improve performance and/or agreement between callers. Here, the focus is on making the filtering criteria of the algorithms as similar as possible. We call these filtering parameter settings our “consistent” or “common” parameter values.

**Fig. 3:**
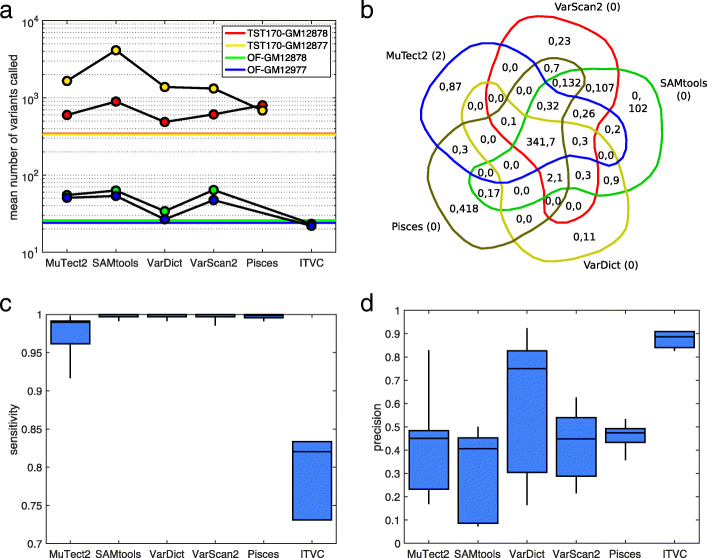
Summary of variant calling across all 18 datasets, when using a consistent set of calling parameters. **a** Numbers of SNVs called by different variant callers, averaged across replicates within our four conditions: GM12878 or GM12877 cells, sequenced using TST170/Illumina or OF/IonTorrent. Colored horizontal lines indicate the number of gold standard mutations that ideally would be detected. **b** Venn diagram of calling results for five callers on replicate one of the TST170–GM12878 data. Within each region, the two numbers give the number of true positive (TP) and false positive (FP) calls agreed upon by the relevant algorithms. The numbers in parentheses next to each algorithm’s name are the number of false negatives (FN) by that algorithm. **c**,**d** Sensitivity (TP/TP+FN) and precision (TP/TP+FP) for each algorithm across all the datasets to which it was applied. The box-and-whisker plots show the percentiles: 0% (i.e. minimum), 25%, 50% (i.e. median), 75%, and 100% (i.e. maximum)

Figure 3 summarizes the results of SNV calling with these common parameters, while Supplementary Tables 5 to 8 give detailed statistics. In Fig. 3a, we see that making parameters consistent greatly reduces the number of variants called by SAMtools–by approximately two orders of magnitude. On the TST170 data, it still calls more SNVs than other algorithms, but on the OF data it is comparable to both MuTect2 and VarScan2. We also see a substantial increase in the number of mutations being called by VarDict. Much of this is due to our lowering of VarDict’s alternative allele frequency threshold from the default value of 0.05 to 0.01. ITVC calls many fewer SNVs than with default parameters, approximately equal to the number of gold standard mutations.

Figure 3b shows a Venn diagram of caller agreement for the same replicate one of TST170–GM12878 data that we showed in Fig. 2b. We continue to see good detection of the gold-standard mutations, but now false positive rates are dramatically reduced—mostly notably for SAMtools. All five algorithms now have unique false positives that are not called by any other algorithm, with Pisces having the most at 418.

Figure 3c,d shows the sensitivity and precision values for the algorithms. With consistent filtering parameters, sensitivity remains fairly high, although MuTect2 has lost some ground, and ITVC even more so. On the other hand, ITVC is now the top peformer in terms of precision, because it makes so many fewer false positive calls. VarDict’s excellent precision with default parameters is lost by adopting the common parameters, although it remains second, on average, after ITVC. Overall, these results highlight several important points. First, a substantial portion of the disagreement between callers that we observed in the previous section can be attributed to differences in default values for things such as minimum allele frequency, coverage, or read quality. Still, significant differences remain between the callers, which may be due to filtering parameters that we could not align, or may be due to differences in their statistical models. And finally, because almost all callers are correctly finding almost all gold-standard mutations, the main differentiator between the algorithms is the false positive calls they make.

### Thresholding depth and allele frequency is inadequate for removing false positives

Variant calls are most ambiguous when allele frequency or coverage are low. Indeed, practitioners in a clinical sequencing laboratory will sometimes visualize coverage and allele frequency in a genome browser to verify variant calls. Although the statistical models employed by the different variant callers are intended to account for allele frequency and coverage, there is always a trade-off between Type I and Type II errors, or in other words, false positive and false negative rates. Because the results in the previous section suggested high sensitivity for detecting mutations, but also high levels of false positives, it seemed plausible that increasing the stringency of variant calls could lead to improved performance.

To formalize the trade-offs involved in coverage and allele frequency, we post-filtered all variant calls at 10 different levels of stringency: a minimum of 20 reads and allele frequency 0.01, 40 reads and allele frequency 0.02, 60 reads and allele frequency 0.03, and so on up to requiring a minimum of 200 reads and allele frequency 0.10. The least stringent threshold is almost equivalent to the results reported in the previous subsection, as our variant calling parameters already included a minimum allele frequency of 0.01, and very few mutations can be called with fewer than 20 reads. The most stringent threshold would be considered unreasonably stringent by many practitioners—firstly, because mutations with allele frequency less than 0.10 can be clinically relevant, and secondly because there is a general consensus that even baseline clinical sequencing approaches ought to be able to detect mutations down to at least 0.05 allele frequency.

**Fig. 4:**
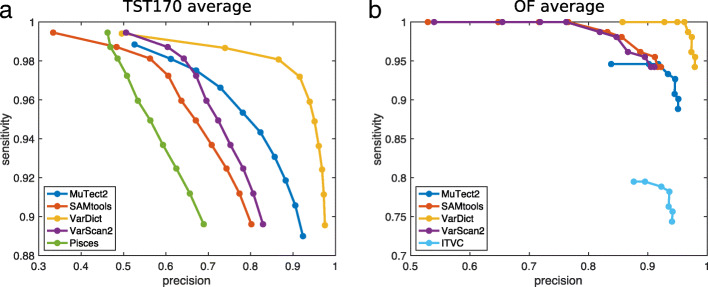
Effect of increasingly stringent alternative allele frequency and coverage thresholds on sensitivity and precision of variant calling. Each curve represents a different algorithm. Each dot on each curve represents a different threshold, ranging from requiring alternative allele frequency AF ≥0.01 and coverage ≥20 reads (least stringent) to AF ≥0.1 and coverage ≥200 reads (most stringent). **a** Averaged results across all TST170 datasets. **b** Averaged results across all OF datasets

Figure 4 shows the sensitivity and precision of each algorithm’s variant calls when post-filtered at different levels of coverage and alternative allele frequency. Panel A shows results averaged across all the TST170 datasets, while panel B shows results averaged across all the OF datasets. Each algorithm has its own curve in the plots, and each curve has 10 circles, corresponding to increasing levels of stringency as one goes rightward and downward. As expected, for all algorithms, increasingly stringent filtering improves precision (or, equivalently, lowers the false discovery rate). VarDict’s results benefit the most, going from 50% and 85% precision on TST170 and OF data respectively, to over 95% precision on both. Other algorithms, while improving precision substantially, still do not reach satisfactory precision levels. For example, even with the most stringent filtering, Pisces and SAMtools achieve only 70% and 80% precision respectively on the TST170 data. False discovery rates of 30% or 20%, respectively, would likely be unacceptable in many clinical sequencing applications.

Stringent filtering also brings with it a notable loss in sensitivity. On the TST170 data, we find that all callers lose approximately 10% of their true positives, when filtered with maximum stringency. On the OF data, 5% of true positives are lost. For most algorithms, moderately stringent filtering—requiring around 0.03 to 0.05 allele frequency and 60 to 100 reads—improves precision with relatively little loss in sensitivity. Overall, however, filtering on allele frequency and coverage does not appear to offer a satisfactory improvement in precision for most algorithms, and even when it does improve precision, it comes with a dissatisfying loss in sensitivity.

### Replicate analysis increases the accuracy of an individual mutation caller

We also studied the possibility of improving performance by analyzing multiple replicates. Few variant callers explicitly handle replicate experiments. However, results from individual replicates can be intersected, or more generally we can use “voting" schemes to combine the answers from multiple replicates [30, 31]. Here, we investigate one straightforward voting scheme. Our OF datasets were each performed in technical triplicate for each of the two cell lines. Our TST170 datasets, six for each cell line, were performed as biological duplicates, and for each biological duplicate as technical triplicates. See Methods for more information. Thus, our data can be naturally divided into six biological experiments, each sequenced in technical triplicate. For each of those six biological experiments, and for each variant caller, we determined whether 1, 2, or all 3 technical replicates detected each possible variant.

**Fig. 5:**
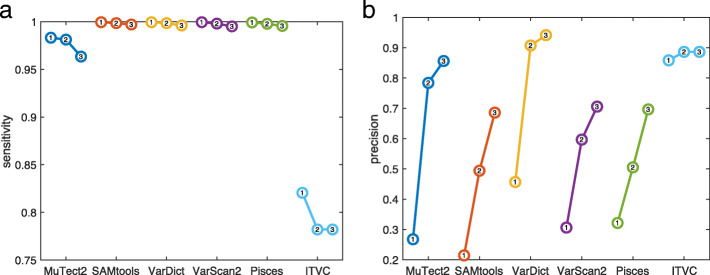
Effect of requiring 1, 2, or 3 replicates to agree on a variant call. **a** Sensitivity averaged across all datasets. **b** Precision averaged across all datasets

Figure 5 shows the sensitivity and precision of the different algorithms when we require that 1, 2, or all 3 replicates find the same variant. Generally, when we require more replicates to show the same variant, we expect that sensitivity may decrease, but precision may increase. Our results confirm this expectation. The particularly good news is that SAMtools, VarDict, VarScan2, and Pisces show only very minor losses in sensitivity, even when all three replicates are required to confirm a variant—remaining at over 99% success in detecting the gold-standard mutations. At the same time, they gain substantially in precision. VarDict in particular achieves approximately 95% precision (5% false discovery rate) while maintaining high sensitivity. MuTect2 also gains much precision, but its sensitivity suffers more. ITVC, which had the lowest sensitivity but highest precision on single-replicate analyses, does not benefit from combining replicates. Overall, then, we find that intersecting or “voting" the results of multiple replicates can greatly reduce false positives with little cost in true positives—unlike the case with allele frequency and coverage thresholding, where false positive reductions were tied to true positive reductions. Still, VarDict is the only algorithm with truly good sensitivity and precision. Even when intersecting calls from three replicate datasets, the other algorithms do not reach 90% precision, and the sensitive algorithms (SAMtools, VarScan2, and Pisces) only reach 30% precision.

### Intersecting mutation callers’ results reduces false positives, while maintaining sensitivity

From Figs. 3 through 5, it is clear that each mutation caller reports many false positives, and that post-filtering results can help eliminate these, but not entirely. When different algorithms produce different results on the same data, a common strategy is to combine or intersect their results [32, 33]. Indeed, our list of known gold-standard mutations was created by Eberle et al. [23] by combining calls made by FreeBayes, Platypus, GATK3, and Strelka. For our data, we were interested in the question of which combination(s) of mutation callers offered the best performance. To test this thoroughly, we computed the SNV calls in the intersection of the calls of every possible combination of algorithms. Because we applied five algorithms to our TST170 data (MuTect2, SAMtools, VarDict, VarScan2, and Pisces), there were 2^5^−1=31 possible combinations of algorithms—comprising five single algorithms (a trivial “combination”), 10 pairs of algorithms (e.g., MuTect2 & SAMtools, or VarDict & VarScan2), 10 trios of allgorithms (e.g., MuTect2, VarDict, & Pisces), five four-way combinations of algorithms, and the single five-way combination of all algorithms. For every combination and for every dataset, we intersected the calls of the algorithms in the combination, and we evaluated sensitivity and precision. We then averaged those results across all the TST170 datasets. We also performed the identical procedure for our OF data, except with MuTect2, SAMtools, VarDict, VarScan2, and ITVC being the five algorithms that we combined in all possible ways.

**Fig. 6:**
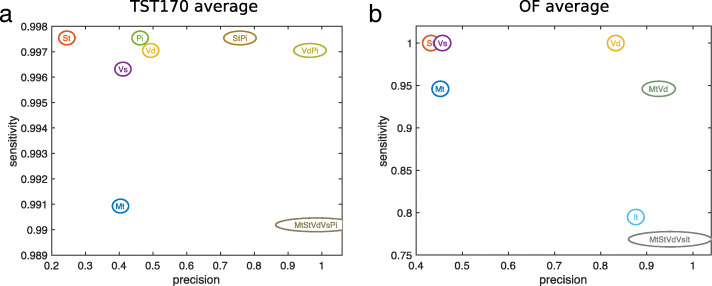
Effect of intersecting the calls from select combinations of algorithms. Each bubble represents either a single algorithm or the intersection of the results of multiple algorithms. For brevity, algorithm names are coded as: Mi = Mutect2, St = SAMtools. Vd = VarDict, Vs = VarScan2, Pi = Pisces, It = ITCV. **a** Averaged results across all TST170 datasets. **b** Averaged results across all OF datasets

Supplementary Tables 9 and 10 give the full results, while Fig. 6 shows the sensitivity and precision of select combinations. For example, on the TST170 data, intersecting the results of SAMtools and Pisces maintains the excellent sensitivity of both algorithms, while boosting precision to over 75%. Intersecting the results of VarDict and Pisces also maintains sensitivity, while boosting precision to over 95%. Generally speaking, when multiple algorithms’ results are intersected, sensitivity either stays the same or goes down—because no additional TPs can be produced, while some may be lost. At the same time, precision has the potential to increase greatly—particularly if different algorithms are committing different false positives. Remarkly, even when we intersect all five algorithms’ results on the TST170 data (Fig. 6a or Supplementary Table 9), we maintain a sensitivity of over 99% while achieving precision of over 98% (i.e. false discovery rate less than 2%). For the OF data, the results are somewhat different. Again, there is a combination of two algorithms that performs very well. However, it is a different pair of algorithms, MuTect2 and VarDict. Adding additional algorithms to the combination does little to further increase precision, and can hurt sensitivity significantly. VarDict alone also performs quite well on the OF data, with perfect 100% sensitivity and precision over 80%. So it is understandable that intersecting additional algorithms has less benefit than we saw in the TST170 data. Still, overall we see that combining results of different callers can significantly benefit precision / false discovery rate, with little to no loss of sensitivity. Intuitively, this means that when variant callers make errors, many of those are different, non-overlapping errors, so that intersecting results can eliminate them.

## Discussion

In this work, we looked at factors contributing to the success of SNV calling in clinical-style samples, including different sequencing platforms, targeting panels, variant callers, and replication. We generally found similar results for the TruSight 170 data sequenced on an Illumina platform and for the Oncomine Focus data sequenced on an Ion Torrent platform. Thus, we concluded that sequencing platform and targeting panel are not major influences on performance, although certainly the two panels offer different coverage of cancer-related genes. We found, as have other groups [12, 15, 34], that different variant callers can disagree widely on the same data. However, we went farther than previous efforts in explaining these differences. First, we found that much can be attributed to various default filtering settings of the algorithms. When making filtering as consistent as possible, disagreements between algorithms shrink considerably. Importantly, all the algorithms that we tested displayed excellent sensitivity for detecting true mutations. The problem is with false positives. Here we found that, using the old bioinformatics strategy of intersecting results, combining results from multiple mutation callers can eliminate many false positives. Other groups have found similar results in variant calling, using simple intersections or more sophisticated combination methods [35, 36]. Importantly, we found that intersecting the results from multiple replicate samples was also a powerful way of filtering out false positives while maintaining detection sensitivity. Although a costlier option than running multiple algorithms, more research should be done into the tradeoff between accuracy and cost of this strategy, and whether it is relevant to clinical practice.

Our study is not without limitations. We have focused on just five variant callers, chosen on the basis of offering tumour-only mode, and being publicly available, free to use, easy to install, and robustly running. Another limitation is that the gold-standard variants we wanted the algorithms to identify were either heterozygous or homozygous in comparison with the hg19 human genome. Thus, their allele frequencies were clustered around 50% or 100%. Detecting mutations at lower allele frequencies can be very important, because real tumor specimens can be heterogeneous. They may mix health and cancer tissue, or they may mix different subclones of the cancer with different mutations—and possibly different drug sensitivities [37]. As such, our finding of nearly-uniform high sensitivity for true mutation detection might not generalize to real tumor specimens with lower mutation allele frequencies, and there may be more challenging trade-offs between sensitivity and precision. Other studies have used mixtures of DNA from different cell lines to “create” mutations at lower allele frequencies (e.g. [13]), and better probe the relationship between allele frequency and detectability. We currently have similar efforts under way. That being said, some of our heterozygous mutations were coming in at apparent allele frequencies as low as 0.1 or 0.2, particularly when coverage was lower. So we were able to derive some understanding of the relationships between coverage, allele frequency and detectability. Another of our long-term goals is to apply similar analyses to real lung cancer specimens in which gold-standard mutations have been identified, so that we can assess detection rates specifically for cancer-relevant mutations in real patient data. Further, we intend to incorporate those results into broader health economic analyses, going beyond mere accuracy to estimate the value of different sequencing platforms, targeting panels, and bioinformatics pipelines to ultimately improving clinical outcomes.

## Conclusions

Clinical sequencing centers face many decisions, including which sequencing technologies and protocols to implement. Our study compared Illumina NextSeq500 and Ion Torrent PGM platforms, and did not find any important difference in accuracy. Similarly, we compared TruSight 170 and Oncomine Focus targeting panels, and found no important difference in accuracy. Thus, although different choices may have different implications regarding cost, coverage, maintenance, or other factors, it is reassuring that accuracy is equally good with any of these choices. We found that different single nucleotide variant calling pipelines could produce highly divergent results, but that much of this is due to differences in default parameters. All pipelines had good sensitivity for detecting mutations, but produced numerous false positive calls. Vendor-recommended pipelines were no better than other pipelines in this regard. However, different pipelines produced different false positives. As well, different false positives were seen in different technical replicates of the data. Thus, we recommend intersecting results from multiple pipelines and/or multiple replicates to minimize false positives.

## Supplementary information

**Additional file 1** Supplementary Material.

## Data Availability

All sequencing data is available from the Sequence Read Archive under project accession PRJNA614006. Human genome version hg19 / GRCh37 is available from the UCSC Genome Browser website (genome.ucsc.edu). Gold-standard variant calls (the authors call them platinum standard) are available through the dbGAP website (https://www.ncbi.nlm.nih.gov/gap/) under accession phs001224.v1.p1.

## Abbreviations

AF: Allele frequencey
FFPE: Formalin-fixed paraffin-embedded
FN: False negative
FNA: Fine needle aspirates
FP: False positive
NGS: Next-generation sequencing
OF: Oncomine focus panel from Thermo fisher scientific
SNV: Single-nucleotide variant
SRA: Sequence read archive
TP: True positive
TST170: TruSight Tumor 170 panel from Illumina
VCF: Variant call file
