# Supplementary material for: Accuracy and reproducibility of somatic point mutation calling in clinical-type targeted sequencing data
Source: BMC Med Genomics. 2020 Oct 15;13:156. doi: 10.1186/s12920-020-00803-z (PMC7560075; doi:10.1186/s12920-020-00803-z)
Supplement: Supplementary file 1 — Additional file 1 Supplementary Material. [file 12920_2020_803_MOESM1_ESM.pdf]

Supplementary Material for:

Accuracy and Reproducibility of Somatic Point Mutation Calling in Clinical-Type Targeted Sequencing Data

Ali Karimnezhad, Gareth A Palidwor, Kednapa Thavorn, David J Stewart, Pearl A Campbell, Bryan Lo, Theodore J. Perkins

Table 1: TST170–GM12878 default parameters

Caller	Rep	NumCalls	TP	FP	FN	Sens.	Prec.
Mutect2	1	514	340	174	3	0.99125	0.66148
	2	477	338	139	5	0.98542	0.7086
	3	416	339	77	4	0.98834	0.8149
	4	721	339	382	4	0.98834	0.47018
	5	769	338	431	5	0.98542	0.43953
	6	766	339	427	4	0.98834	0.44256
Samtools	1	325816	342	325474	1	0.99708	0.0010497
	2	127858	341	127517	2	0.99417	0.002667
	3	206361	341	206020	2	0.99417	0.0016524
	4	145498	342	145156	1	0.99708	0.0023505
	5	136664	342	136322	1	0.99708	0.0025025
	6	138410	342	138068	1	0.99708	0.0024709
VarDict	1	347	342	5	1	0.99708	0.98559
	2	355	340	15	3	0.99125	0.95775
	3	351	341	10	2	0.99417	0.97151
	4	346	342	4	1	0.99708	0.98844
	5	344	341	3	2	0.99417	0.99128
	6	345	342	3	1	0.99708	0.9913
VarScan2	1	729	342	387	1	0.99708	0.46914
	2	552	339	213	4	0.98834	0.61413
	3	593	340	253	3	0.99125	0.57336
	4	662	342	320	1	0.99708	0.51662
	5	629	342	287	1	0.99708	0.54372
	6	639	342	297	1	0.99708	0.53521
Pisces	1	1055	342	713	1	0.99708	0.32417
	2	773	341	432	2	0.99417	0.44114
	3	783	341	442	2	0.99417	0.4355
	4	907	342	565	1	0.99708	0.37707
	5	949	342	607	1	0.99708	0.36038
	6	1045	342	703	1	0.99708	0.32727

Table 2: TST170–GM12877 default parameters

Caller	Rep	NumCalls	TP	FP	FN	Sens.	Prec.
Mutect2	1	1903	334	1569	2	0.99405	0.17551
	2	1773	335	1438	1	0.99702	0.18895
	3	1426	332	1094	4	0.9881	0.23282
	4	1330	335	995	1	0.99702	0.25188
	5	2036	334	1702	2	0.99405	0.16405
	6	1626	335	1291	1	0.99702	0.20603
Samtools	1	50468	336	50132	0	1	0.0066577
	2	46565	336	46229	0	1	0.0072157
	3	42572	335	42237	1	0.99702	0.007869
	4	62048	336	61712	0	1	0.0054152
	5	71143	336	70807	0	1	0.0047229
	6	62740	336	62404	0	1	0.0053554
VarDict	1	355	336	19	0	1	0.94648
	2	353	336	17	0	1	0.95184
	3	364	335	29	1	0.99702	0.92033
	4	351	336	15	0	1	0.95726
	5	358	335	23	1	0.99702	0.93575
	6	361	336	25	0	1	0.93075
VarScan2	1	1242	335	907	1	0.99702	0.26973
	2	1246	334	912	2	0.99405	0.26806
	3	1138	334	804	2	0.99405	0.2935
	4	1758	336	1422	0	1	0.19113
	5	1651	336	1315	0	1	0.20351
	6	1589	335	1254	1	0.99702	0.21082
Pisces	1	809	336	473	0	1	0.41533
	2	813	336	477	0	1	0.41328
	3	732	335	397	1	0.99702	0.45765
	4	883	336	547	0	1	0.38052
	5	883	336	547	0	1	0.38052
	6	827	336	491	0	1	0.40629

Table 3: OF–GM12878 default parameters

Caller	Rep	NumCalls	TP	FP	FN	Sens.	Prec.
Mutect2	1	55	25	30	1	0.96154	0.45455
	2	48	26	22	0	1	0.54167
	3	41	26	15	0	1	0.63415
Samtools	1	21578	26	21552	0	1	0.0012049
	2	12704	26	12678	0	1	0.0020466
	3	9171	26	9145	0	1	0.002835
VarDict	1	27	26	1	0	1	0.96296
	2	26	25	1	1	0.96154	0.96154
	3	27	26	1	0	1	0.96296
VarScan2	1	67	26	41	0	1	0.38806
	2	145	26	119	0	1	0.17931
	3	238	26	212	0	1	0.10924
ITVC	1	315	25	290	1	0.96154	0.079365
	2	315	25	290	1	0.96154	0.079365
	3	314	25	289	1	0.96154	0.079618

Table 4: OF–GM12877 default parameters

Caller	Rep	NumCalls	TP	FP	FN	Sens.	Prec.
Mutect2	1	40	22	18	2	0.91667	0.55
	2	52	23	29	1	0.95833	0.44231
	3	56	22	34	2	0.91667	0.39286
Samtools	1	17770	24	17746	0	1	0.0013506
	2	20949	24	20925	0	1	0.0011456
	3	24386	24	24362	0	1	0.00098417
VarDict	1	23	23	0	1	0.95833	1
	2	24	23	1	1	0.95833	0.95833
	3	24	23	1	1	0.95833	0.95833
VarScan2	1	49	24	25	0	1	0.4898
	2	53	24	29	0	1	0.45283
	3	50	24	26	0	1	0.48
ITVC	1	325	23	302	1	0.95833	0.070769
	2	325	23	302	1	0.95833	0.070769
	3	325	23	302	1	0.95833	0.070769

Table 5: TST170–GM12878 consistent parameters

Caller	Rep	NumCalls	TP	FP	FN	Sens.	Prec.
Mutect2	1	502	341	161	2	0.99417	0.67928
	2	472	337	135	6	0.98251	0.71398
	3	409	339	70	4	0.98834	0.82885
	4	703	340	363	3	0.99125	0.48364
	5	755	340	415	3	0.99125	0.45033
	6	754	340	414	3	0.99125	0.45093
Samtools	1	784	343	441	0	1	0.4375
	2	1354	340	1014	3	0.99125	0.25111
	3	1079	342	737	1	0.99708	0.31696
	4	700	343	357	0	1	0.49
	5	731	343	388	0	1	0.46922
	6	715	343	372	0	1	0.47972
VarDict	1	378	343	35	0	1	0.90741
	2	679	340	339	3	0.99125	0.50074
	3	546	342	204	1	0.99708	0.62637
	4	415	343	72	0	1	0.82651
	5	465	342	123	1	0.99708	0.73548
	6	435	343	92	0	1	0.78851
VarScan2	1	685	343	342	0	1	0.50073
	2	540	338	202	5	0.98542	0.62593
	3	580	341	239	2	0.99417	0.58793
	4	636	343	293	0	1	0.53931
	5	605	343	262	0	1	0.56694
	6	615	343	272	0	1	0.55772
Pisces	1	961	343	618	0	1	0.35692
	2	707	340	367	3	0.99125	0.48091
	3	699	342	357	1	0.99708	0.48927
	4	804	343	461	0	1	0.42662
	5	849	343	506	0	1	0.404
	6	780	343	437	0	1	0.43974

Table 6: TST170–GM12877 consistent parameters

Caller	Rep	NumCalls	TP	FP	FN	Sens.	Prec.
Mutect2	1	1873	333	1540	3	0.99107	0.17779
	2	1751	333	1418	3	0.99107	0.19018
	3	1422	331	1091	5	0.98512	0.23277
	4	1297	335	962	1	0.99702	0.25829
	5	1978	334	1644	2	0.99405	0.16886
	6	1595	334	1261	2	0.99405	0.2094
Samtools	1	3909	335	3574	1	0.99702	0.0857
	2	4419	334	4085	2	0.99405	0.075583
	3	4494	334	4160	2	0.99405	0.074321
	4	3883	336	3547	0	1	0.086531
	5	3471	336	3135	0	1	0.096802
	6	4571	335	4236	1	0.99702	0.073288
VarDict	1	1406	335	1071	1	0.99702	0.23826
	2	1518	334	1184	2	0.99405	0.22003
	3	2038	334	1704	2	0.99405	0.16389
	4	1164	336	828	0	1	0.28866
	5	1056	335	721	1	0.99702	0.31723
	6	1101	335	766	1	0.99702	0.30427
VarScan2	1	1162	335	827	1	0.99702	0.2883
	2	1194	333	861	3	0.99107	0.27889
	3	1073	333	740	3	0.99107	0.31034
	4	1567	336	1231	0	1	0.21442
	5	1485	336	1149	0	1	0.22626
	6	1428	335	1093	1	0.99702	0.23459
Pisces	1	676	335	341	1	0.99702	0.49556
	2	655	334	321	2	0.99405	0.50992
	3	626	334	292	2	0.99405	0.53355
	4	720	336	384	0	1	0.46667
	5	743	336	407	0	1	0.45222
	6	689	335	354	1	0.99702	0.48621

Table 7: OF–GM12878 consistent parameters

Caller	Rep	NumCalls	TP	FP	FN	Sens.	Prec.
Mutect2	1	55	25	30	1	0.96154	0.45455
	2	55	25	30	1	0.96154	0.45455
	3	55	25	30	1	0.96154	0.45455
Samtools	1	63	26	37	0	1	0.4127
	2	63	26	37	0	1	0.4127
	3	63	26	37	0	1	0.4127
VarDict	1	34	26	8	0	1	0.76471
	2	34	26	8	0	1	0.76471
	3	34	26	8	0	1	0.76471
VarScan2	1	64	26	38	0	1	0.40625
	2	64	26	38	0	1	0.40625
	3	64	26	38	0	1	0.40625
ITVC	1	23	19	4	7	0.73077	0.82609
	2	25	21	4	5	0.80769	0.84
	3	22	19	3	7	0.73077	0.86364

Table 8: OF–GM12877 consistent parameters

Caller	Rep	NumCalls	TP	FP	FN	Sens.	Prec.
Mutect2	1	41	22	19	2	0.91667	0.53659
	2	53	23	30	1	0.95833	0.43396
	3	58	22	36	2	0.91667	0.37931
Samtools	1	53	24	29	0	1	0.45283
	2	60	24	36	0	1	0.4
	3	48	24	24	0	1	0.5
VarDict	1	27	24	3	0	1	0.88889
	2	27	24	3	0	1	0.88889
	3	26	24	2	0	1	0.92308
VarScan2	1	46	24	22	0	1	0.52174
	2	49	24	25	0	1	0.4898
	3	47	24	23	0	1	0.51064
ITVC	1	22	20	2	4	0.83333	0.90909
	2	22	20	2	4	0.83333	0.90909
	3	22	20	2	4	0.83333	0.90909

Table 9: Average sensitivity and precision on TST170 data when calls from different combinations of algorithms are intersected

Combination	Sensitivity	Precision
VarDict	0.99705	0.49311
MuTect2	0.99093	0.40369
Pisces	0.99754	0.4618
SAMtools	0.99754	0.24473
VarScan2	0.99632	0.41095
VarDict Pisces	0.99705	0.96484
VarDict VarScan2	0.99582	0.84388
MuTect2 VarDict	0.99093	0.75731
MuTect2 Pisces	0.99093	0.94935
MuTect2 SAMtools	0.99093	0.8493
MuTect2 VarScan2	0.99019	0.86984
SAMtools VarDict	0.99705	0.62907
SAMtools Pisces	0.99754	0.75762
SAMtools VarScan2	0.99632	0.44677
VarScan2 Pisces	0.99632	0.78136
VarDict VarScan2 Pisces	0.99582	0.97315
MuTect2 VarDict Pisces	0.99093	0.9823
MuTect2 VarDict VarScan2	0.99019	0.94007
MuTect2 SAMtools VarDict	0.99093	0.92933
MuTect2 SAMtools Pisces	0.99093	0.95357
MuTect2 SAMtools VarScan2	0.99019	0.87255
MuTect2 VarScan2 Pisces	0.99019	0.9538
SAMtools VarDict Pisces	0.99705	0.96968
SAMtools VarDict VarScan2	0.99582	0.84519
SAMtools VarScan2 Pisces	0.99632	0.79227
MuTect2 VarDict VarScan2 Pisces	0.99019	0.98277
MuTect2 SAMtools VarDict Pisces	0.99093	0.98301
MuTect2 SAMtools VarDict VarScan2	0.99019	0.94115
MuTect2 SAMtools VarScan2 Pisces	0.99019	0.95421
SAMtools VarDict VarScan2 Pisces	0.99582	0.97361
MuTect2 SAMtools VarDict VarScan2 Pisces	0.99019	0.98324

Table 10: Average sensitivity and precision on OF data when calls from different combinations of algorithms are intersected

Combination	Sensitivity	Precision
VarDict	1	0.8325
MuTect2	0.94605	0.45225
ITVC	0.79487	0.87617
SAMtools	1	0.43182
VarScan2	1	0.45682
VarDict ITVC	0.79487	0.93749
VarDict VarScan2	1	0.83819
MuTect2 VarDict	0.94605	0.92585
MuTect2 ITVC	0.7687	0.88519
MuTect2 SAMtools	0.94605	0.86473
MuTect2 VarScan2	0.94605	0.86473
SAMtools VarDict	1	0.84389
SAMtools ITVC	0.79487	0.87617
SAMtools VarScan2	1	0.47836
VarScan2 ITVC	0.79487	0.87617
VarDict VarScan2 ITVC	0.79487	0.93749
MuTect2 VarDict ITVC	0.7687	0.95036
MuTect2 VarDict VarScan2	0.94605	0.92585
MuTect2 SAMtools VarDict	0.94605	0.92585
MuTect2 SAMtools ITVC	0.7687	0.88519
MuTect2 SAMtools VarScan2	0.94605	0.86473
MuTect2 VarScan2 ITVC	0.7687	0.88519
SAMtools VarDict ITVC	0.79487	0.93749
SAMtools VarDict VarScan2	1	0.84389
SAMtools VarScan2 ITVC	0.79487	0.87617
MuTect2 VarDict VarScan2 ITVC	0.7687	0.95036
MuTect2 SAMtools VarDict ITVC	0.7687	0.95036
MuTect2 SAMtools VarDict VarScan2	0.94605	0.92585
MuTect2 SAMtools VarScan2 ITVC	0.7687	0.88519
SAMtools VarDict VarScan2 ITVC	0.79487	0.93749
MuTect2 SAMtools VarDict VarScan2 ITVC	0.7687	0.95036
